# CDKN2BAS gene polymorphisms and the risk of intracranial aneurysm in the Chinese population

**DOI:** 10.1186/s12883-017-0986-z

**Published:** 2017-12-11

**Authors:** Yunchang Chen, Gancheng Li, Haiyan Fan, Shenquan Guo, Ran Li, Jian Yin, Xin Zhang, Xifeng Li, Xuying He, Chuanzhi Duan

**Affiliations:** 1 Department of Neurosurgery, The National Key Clinic Specialty, The Neurosurgery Institute of Guangdong Province, Guangdong Provincial Key Laboratory on Brain Function Repair and Regeneration, Zhujiang Hospital, Southern Medical University, Guangzhou, 510282 China; 2 Department of Neurosurgery, Southern Medical University, Zhujiang Hospital, 253# industry road, Guangzhou, Guangdong 510282 China

**Keywords:** CDKN2BAS gene, Polymorphisms, Intracranial aneurysms

## Abstract

**Background:**

CDKN2BAS gene polymorphisms has been shown to correlation with intracranial aneurysm(IA) in the study of foreign people. The study, the author selected the Chinese people as the research object to explore whether CDKN2BAS gene polymorphisms associated with Chinese patients with IA.

**Methods:**

We selected 200 patients(52.69 ± 11.50) with sporadic IA as experimental group, 200 participants(49.99 ± 13.00) over the same period to the hospital without cerebrovascular diseases as control group. Extraction of peripheral blood DNA, applying polymerase chain reaction(PCR)-ligase detection reaction (LDR) identified CDKN2BAS Single nucleotide polymorphism(SNP) locus genotype: rs6475606, rs1333040, rs10757272, rs3217992, rs974336, rs3217986, rs1063192. The differences in allelic and genotype frequencies between the patient and control groups were evaluated by the chi-square test or Fisher’s exact tests.

**Results:**

The genotype of rs1333040 and rs6475606 shown association with sporadic IA(X^2^ = 8.545, *P* = 0.014; X^2^ = 10.961, *P* = 0.004; respectively);the C allele of rs6475606 showed reduction the occurrence of IA; the rs1333040 and rs6475606 associated with hemorrhage, the C allele of rs1333040 could lower the risk of hemorrhage, and rs6475606 will not, rs1333040 also associated with aneurysm size.

**Conclusion:**

Our research shows that variant rs1333040 and rs6475606 of CDKN2BAS related to the Chinese han population of sporadic IAs occurs. This study confirms the association between CDKN2BAS and IAs.

## Background

IA is a complex disease affecting human health, it is the primary cause of subarachnoid hemorrhage. According to statistics, in the event of rupture of IA, patients’ mortality and morbidity is 30 and 25%, respectively. It is generally accepted that IA influenced both by genetic and environmental risk factors. Environmental factors including smoking, drinking, hypertension, diabetes and so on. Genetic factors, there are a number of human genes associated with the occurrence and development of IA [1, 2, 22]. Genome-wide association study(GWAS) as a kind of method research on diseases, which applied to study of IA has found associated with these chromosomes:4q(EDNRA),5q31.3,6q24.2,8q12.1(SOX17), 9p21.3(CDKN2A/CDKN2B/CDKN2BAS),10q24.32(CNNM2),12q22,13q13.1(KL/STARD13), 18q11.2(RBBP8), 20p2.1 [3, 7, 9, 10, 17, 20, 21].

CDKN2BAS located in chromosome 9p21, a 3.8 kb long non-coding RNA(lncRNA) expressed in the opposite direction from INK4A-ARF-INK4B gene cluster, it is associated with a variety of human diseases, such as prostate cancer, stomach cancer, pancreatic cancer, leukemia, glioma, colorectal cancer, lung cancer, diabetes and aneurysm [4, 5, 14, 16]. There has been a lot of scholar studies supported CDKN2BAS gene multiple SNPs loci associated with IA. Hashikata confirmed rs1333040 associated with familial and sporadic IA in Japanese [8]. Foroud and his colleagues study United States population also confirmed CDKN2BAS SNP loci could lead to susceptibility of IA on the basis of predecessors’ research results, and believe rs6475606 have significant effect on IA [6]; another research on Japanese found that rs10757272 is a new susceptibility locus of IA, and confirmed rs10757278 associated with IA [13]; other chromosome gene polymorphism loci showed association with intracranial aneurysm, such as rs6841581(4q), rs12411886(10q), rs6538595(12q), rs9315204(13q), but the CDKN2BAS SNP loci were replicated successfully the most time in later studies [12, 21]. Due to the limitation of the study sample size, these studies credibility is not so high, the relationship between CDKN2BAS SNP loci and IA needs more study.

Whether CDKN2BAS SNP loci associated with China IAs patients has not been reported. Taking into account the same gene mutation rates is differ greatly in different populations and the genetic heterogeneity between races of people, so we use the database to find the minor allele frequency(MAF ≥ 0.05) of CDKN2BAS SNP loci at an r^2^ > 0.8 threshold in Chinese population, including rs3217992, rs974336, rs3217986, rs1063192, explore CDKN2BAS SNP effect on IAs, hope to provide new strategies for prevention and treatment of IAs.

## Methods

### The research object

This study was supported by Zhujiang Hospital of Southern Medical University ethics com- mittee, all the participants reported themselves to be of Han residents, have signed informed consent. Zhujiang Hospital is located in developed areas of China—Guangzhou, where the people from other provinces of China accounts for a large part of city population, and popul- ation flow is also very frequent, so the subjects of this study are representative. All particip- ants exclude genetic history, family history and history of malignancy, controlling the age between 18 and 80 years.

Within 14 months, this study recruited 200 patients with sporadic IAs, the median age was 52.69 ± 11.50 years. Patients with IAs diagnosed by computed tomography angiography(CTA), magnetic resonance angiography(MRA) or digital subtraction angiography(DSA) has at least one aneurysm. For the diagnosis of hemorrhage caused by aneurysms, especially ruptured IAs, not only combined with clinical symptoms, but also confirmed by the DSA results eventually. All cases with confirmed IAs based on DSA were retrospectively reviewed by 2 independent doctors, a neuroradiologist and a neurosurgeon, they are all experienced in the diagnosis of aneurysms. All patients in the study underwent either craniotomy or interventional therapy to confirm the correct diagnosis of IAs again.

During the same period, 200 blood donors form a control group for the study, with a median age of 49.99 ± 13.00. Considering the prevalence of IAs vary between 0.2 and 9% of the population, this study does not recruit volunteers from the outside, control participants were patients(such as cerebral hemorrhage, cerebral trauma, epilepsy) from the Department of Neurosurgery of Zhujiang Hospital, as most of them have imaging data(computed tomography(CT), magnetic resonance imaging(MRI), MRA) and clinical information excluded the presence of aneurysms. Controls were eliminate diseases not only including polycystic kidney disease, Malaysia’s syndrome, systemic lupus erythematosus (SLE), etc., but also severity of neurological diseases.

The subjects fully considered the balance and comparable of the baseline data during collection process, genomic DNA was extracted from Peripheral whole blood use kit, using agarose gel electrophoresis to detect the DNA integrity, ensure the follow-up test smoothly.

### Genotyping and SNP selection of CDKN2BAS

Through foreign research literatures found the highest positive sites correlation with IAs, they are rs1333040, rs6475606, rs10757272; and using genetic database Hapmap (http://hapmap.org), Haploview (http://www.broad.mit.edu/mpg/haploview) found the tagSNP loci of CDKN2BAS in Chinese population, including rs3217992, rs974336, rs3217986, rs1063192; so the seven sites as this research objects, SNP genotyping by PCR-LDR in Shanghai Biowing Applied Biotechnology Co., Ltd. The seven SNPs primer sequences and PCR length are given in Table 1; these SNPs probe sequences and LDR length are read in Table 2. PCR and LDR manipulated as previous describe [18], Firstly, PCR amplification of SNP sites in 20ul master mix, containing 2ul 1 × buffer, 0.6ul Mg++, 2ul dNTPs, 0.2ul Taq polymerase, 4ul 1 × Q-solution, 0.4ul primer mix and 9.8ul ddH^2^O, after thoroughly incorporated, take 19ul partial shipments in PCR reaction tube and add 1ul genomic DNA of samples, set the following program on Gene Amp PCR system (model 9600, Perkin Elmer): denaturing at 95 °C for 15 min; then denaturing at 94 °C for 30 s, annealing at 56 °C for 1 min, extension at 72 °C for 1 min, repeat 35 cycles; also need to extend 7 min under 72 °C temperature. Secondly, LDR performed to further amplification in 10ul volume of Multiplex LDR mixture, before operation LDR add the same as volume ddH^2^O to PCR amplification product, the LDR mixture containing resultant PCR product of 1ul, 1ul probe mix, 0.05ul Taq DNA ligase, and 6.95ul ddH^2^O, after thoroughly incorporated, take 9ul partial shipments in PCR reaction tube and add 1ul PCR amplification product, set LDR conditions: initial denaturing at 95 °C for 2 min, followed by 35 cycles of denaturing at 94 °C for 30 s, and annealing at 50 °C for 2 min, mix 1ul of LDR product with 0.6ul ROX(ABI.GS-500), after denaturing at 95 °C for 2 min, in the ice cold snap, following capillary electrophoresis, meanwhile applying Genemapper(ABI, Inc.) for data analysis and genotyping.

**Table 1:** Primer sequences and PCR length

Primer name	sequence(5-3) up	low	PCR length
rs1333040	TAATGGGATGGAGTGCAGGG	AACCTCCATGAGAACTGGCT	193
rs3217986	CTCCACCAGATAGCAGAGGG	CCACCACCTCATCCTGTGTA	176
rs10757272	CCTGAGGAGAAGAGAAGGGC	TGGGGCACATGATTCCAAAA	180
rs6475606	AAAGGAAGAACATGGCACCC	TTGTAGAACACAACAACCCC	87
rs1063192	GTGTAATATAGTACTGTGGG	CTTGTGGAATCTTTCCTAAT	85
rs974336	CAGACATCAGAGACCTGAAC	GCAGGTGGAGCCATTTAAAG	78
rs3217992	GGAATTAATTTTTACATGGC	TGGGTATCAATTACCACCTG	67

**Table 2:** Probe sequences and LDR length

Probename	sequence(5-3)	LDR length
rs1333040_modify	P-CATTCTTACCTCTGACCCTCTTTTTTTTTTTTTTTTTT-FAM	
rs1333040_C	TTTTTTTTTTTTTTTTCTTCCTCTCTGTCCCAGCGGTAG	77
rs1333040_T	TTTTTTTTTTTTTTTTTTCTTCCTCTCTGTCCCAGCGGTAA	79
rs3217986_modify	P-CCTCTCTTACCCCTCTGCTATTTTTTTTTTTTTTTTTTTTT-FAM	
rs3217986_A	TTTTTTTTTTTTTTTTTTAGCATCTGTCGTCGCTTGCACAT	82
rs3217986_C	TTTTTTTTTTTTTTTTTTTTAGCATCTGTCGTCGCTTGCACAG	84
rs10757272_modify	P-CTTCTTACGACCTTTCATAATTTTTTTTTTTTTTTTTTTTTTTT-FAM	
rs10757272_C	TTTTTTTTTTTTTTTTTTTTATAATAAAACATTGCAACATTCG	87
rs10757272_T	TTTTTTTTTTTTTTTTTTTTTTATAATAAAACATTGCAACATTCA	89
rs6475606_modify	P-CTTTCACTGAGTGTCCATTATTTTTTTTTTTTTTTTTTTTTTTTTTT-FAM	
rs6475606_C	TTTTTTTTTTTTTTTTTTTTTTCCAAAAATGATGATGATAGCCAG	92
rs6475606_T	TTTTTTTTTTTTTTTTTTTTTTTTCCAAAAATGATGATGATAGCCAA	94
rs1063192_modify	P-GTTGTCATTAGGAAAGATTCTTTTTTTTTTTTTTTTTTTTTTTTTTTTTT-FAM	
rs1063192_C	TTTTTTTTTTTTTTTTTTTTTTTTTCTTTAGTTTCCCTTAATATCAG	97
rs1063192_T	TTTTTTTTTTTTTTTTTTTTTTTTTTTCTTTAGTTTCCCTTAATATCAA	99
rs974336_modify	P-AAAGTTTTCACCCAGTGCAGTTTTTTTTTTTTTTTTTTTTTTTTTTTTTTTTT-FAM	
rs974336_A	TTTTTTTTTTTTTTTTTTTTTTTTTTCATTTAAAGAAACACCTAATTGT	102
rs974336_G	TTTTTTTTTTTTTTTTTTTTTTTTTTTTCATTTAAAGAAACACCTAATTGC	104
rs3217992_modify	P-AATACAACCAGGTGGTAATTTTTTTTTTTTTTTTTTTTTTTTTTTTTTTTTTTTTT-FAM	
rs3217992_A	TTTTTTTTTTTTTTTTTTTTTTTTTTTTTGGCATTGATAAGTTACTATTTT	107
rs3217992_G	TTTTTTTTTTTTTTTTTTTTTTTTTTTTTTTGGCATTGATAAGTTACTATTTC	109

### Statistical analysis

Hardy-Weinberg equilibrium tested by chi-square in control, allelic and genotype frequencies are evaluated by chi-square test or Fisher’s exact tests, all data analysis using SPSS 20.0, considering statistical differences when *P* value is less than 0.05.

## Results

### Clinical background of patients

The clinical information of the study participants was summarized in Table 3. Female patients with IAs accounted for 61.5%, is 1.60 times of male patients, and consistent with other related reports [18, 23]. The baseline data balanced between the experimental group and the control group, including gender, smoking, drinking, hypertension and diabetes. Patients with IAs also make specific statistic about aneurysm number and position. There were 90 patients with IAs located in the posterior communicating artery and internal carotid artery, 41 patients in the anterior cerebral artery and 32 patients in posterior cerebral artery. The numbers of patients with one aneurysm, two aneurysms, three aneurysms, more than 3 aneurysms are 130, 35, 21, 14 respectively.

**Table 3:** Characteristics of patients with IAs vs. controls

	Aneurysms group	Controls group	X^2^ value	*P* value
Number	200	200		
Male	77(38.5)	78(39.0)		
Female	123(61.5)	122(61.0)	0.011	0.918
Age	52.69 ± 11.50	49.99 ± 13.00	1.639	0.201
Smoking	43(21.5)	40(20.0)		
Non-smoking	157(78.5)	160(80.0)	0.137	0.711
Drinking	44(22.0)	42(21.0)		
Non-drinker	156(78.0)	158(79.0)	0.059	0.808
Hypertension	59(29.5)	54(27.0)		
Non-hypertension	141(70.5)	146(73.0)	0.308	0.579
Diabetes	24(12.0)	22(11.0)		
Non-diabetes	176(88.0)	178(89.0)	0.098	0.754
Site of aneurysm(%)				
ACA	41(20.5)			
PCA	32(16.0)			
ACoA	16(8.0)			
ICA,PCoA	90(45.0)			
MCA	10(5.0)			
other	11(5.5)			
Number of aneurysm(319)				
One	130(65.0)			
Two	35(17.5)			
Three	21(10.5)			
> three	14(7.0)			

*ACA* anterior cerebral artery, *ACoA* anterior communicating artery, *ICA* internal carotid artery, *MCA* middle cerebral artery, *PCA* posterior cerebral artery, *PCoA* posterior communicating artery

### Allelic association study with SNPs of CDKN2BAS

**Fig. 1:**
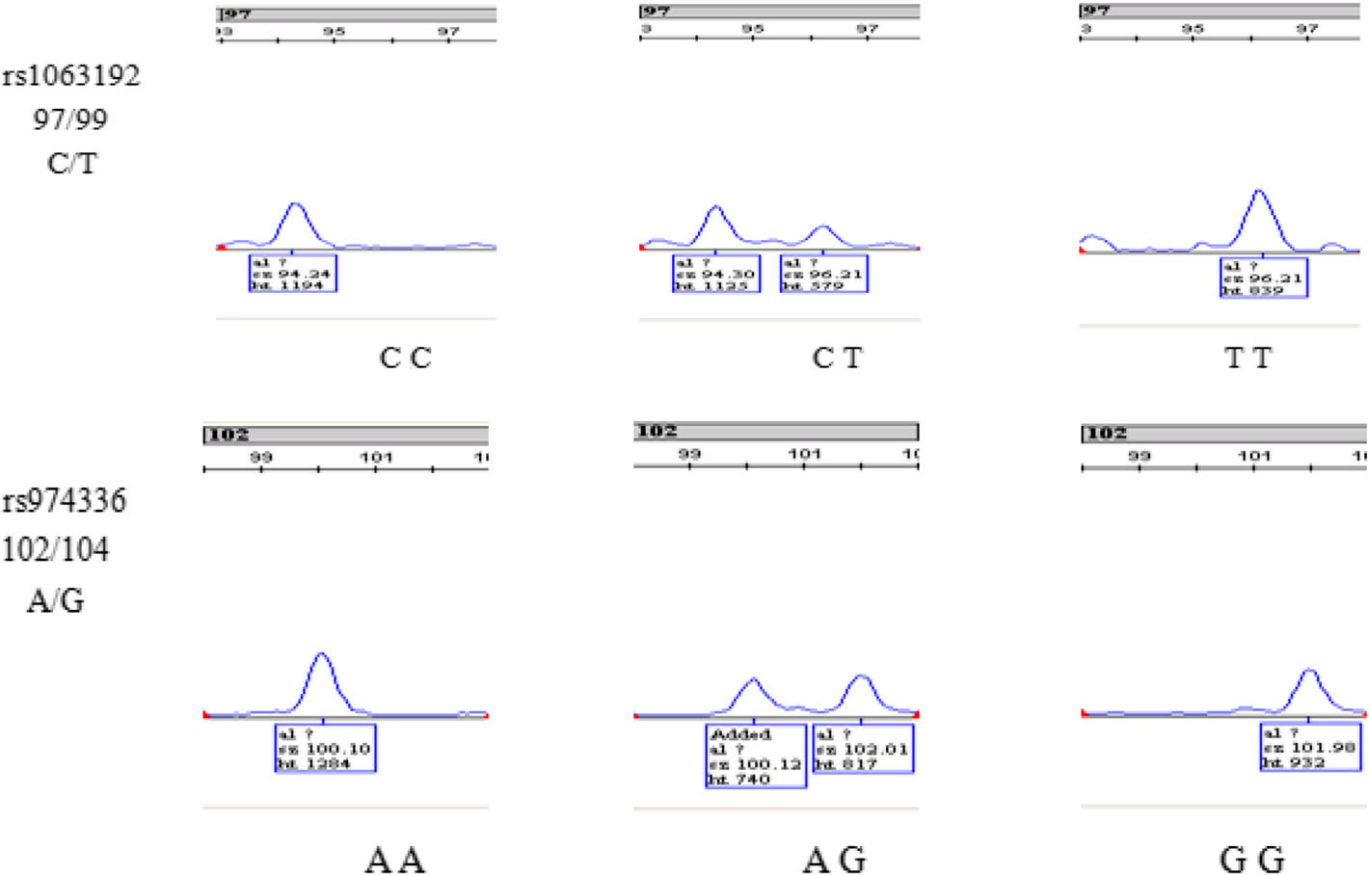
Two loci of the gene-picture

We randomly selected two loci of the Genemapper as shown in Fig. 1. The genotype and allele frequency of cases and controls was presented in Table 4. The control group completely accorded with Hardy-Weinberg equilibrium and objects included in the study could represent the crowd finely. The difference in seven sites of genotype and allele frequency was compared with chi-square or Fisher’s exact tests. The genotype frequency of rs6475606 show significant difference between cases and controls(X^2^ = 10.961, *P* = 0.004), There also existed difference among groups when compared frequency of TT with CC and CT genotypes combined (X^2^ = 9.008, OR 1.831 [95% confidence interval 1.232—2.723], *P* = 0.003), the C allele showed reduction the risk of IAs(X^2^ = 4.784, OR 0.710 [95% confidence interval 0.522—0.966], *P* = 0.029). The distribution of the variants rs1333040 was significantly different in genotype frequency among cases and controls(X^2^ = 8.545, *P* = 0.014), and compared frequency of TT with CC and CT genotypes combined, the results were significant(X^2^ = 7.295, OR 1.722 [95% confidence interval 1.159—2.558], *P* = 0.007).

**Table 4:** genotype and allele frequencies of patients with IAs vs. controls

		Aneurysms	Controls	X^2^ value	*P* value	OR(95%CI)
rs1333040	CC	19(9.5)	18(9)	8.545	0.014	
	CT	65(32.5)	92(46)			
	TT	116(58)	90(45)			
	CC + CT	84(42)	111(55.5)	7.295	0.007	1.722(1.159—2.558)
C allele		103/297(25.75)	128/272(32)	3.804	0.051	0.737(0.542—1.002)
rs3217986	AA	166(83)	157(78.5)	1.597	0.445	
	AC	32(16)	39(19.5)			
	CC	2(1)	4(2)			
	AC + CC	34(17)	43(21.5)	1.303	0.254	0.748(0.454—1.233)
rs10757272	CC	21(10.5)	22(11)	0.497	0.780	
	CT	97(48.5)	90(45)			
	TT	82(41)	88(44)			
	CC + CT	112(56)	111(55.5)	0.368	0.544	1.131(0.760—1.681)
rs6475606	CC	19(9.5)	17(8.5)	10.961	0.004	
	CT	63(31.5)	95(47.5)			
	TT	118(59)	88(44)			
	CC + CT	82(41)	112(56)	9.008	0.003	1.831(1.232—2.723)
C allele		101/299(25.25)	129/271(32.25)	4.784	0.029	0.710(0.522—0.966)
rs1063192	CC	6(3)	5(2.5)	0.783	0.676	
	CT	56(28)	49(24.5)			
	TT	138(69)	146(73)			
	CC + CT	62(31)	54(27)	0.777	0.378	1.215(0.788—1.872)
rs974336	AA	9(4.5)	3(1.5)	3.983	0.137	
	AG	63(31.5)	74(37)			
	GG	128(64)	123(61.5)			
	AA + AG	72(36)	77(38.5)	0.267	0.605	0.899(0.599—1.348)
rs3217992	AA	61(30.5)	60(30)	1.021	0.600	
	AG	101(50.5)	94(47)			
	GG	38(19)	46(23)			
	AG + GG	139(69.5)	140(70)	0.012	0.913	0.977(0.637—1.496)

*OR* odds ratio, *CI* confidence intervals, *P* value was considered significant at a level of <0.05

### Genotype is related to clinical manifestation

The study aimed to assess the correlation between CDKN2BAS SNP genotypes and clinical presentation(such as hemorrhage, epilepsy, aneurysm location and size) of aneurysms(Table 5). Statistical analysis indicated that both of rs1333040 and rs6475606 were associated with hemorrhage. The C allele of rs1333040 reduced the incidence of hemorrhage. The C allele of rs6475606 was overrepresented in controls which controversial with it was the risk of hemorrhage(X^2^ = 9.626, OR 2.002 [95% confidence interval 1.286—3.119], *P* = 0.002); and rs13330 40 also related to aneurysm size(*P* < 0.000), the C allele was the risk factor of IAs with size < 3 cm(X^2^ = 7.358, OR 2.020 [95% confidence interval 1.207—3.380], *P* = 0.007). The study did not find that CDKN2BAS SNP genotypes was associated with epilepsy and aneurysm location.

**Table 5:** Analysis of genotype and clinical presentation

		Hemorrhage (136 patients)	No Hemorrhage (64 patients)	X^2^ value	*P* value	OR(95%CI)
rs1333040	CC	14(10.29)	5(7.80)			
	CT	31(22.79)	34(53.13)	18.421	0.000^*^	
	TT	91(66.92)	25(39.07)			
C allele		59/272(21.69)	44/128(34.38)	4.182	0.041	0.631(0.405—0.983)
rs6475606	CC	13(9.56)	6(9.38)			
	CT	26(19.12)	37(57.81)	31.628	0.000^*^	
	TT	97 (71.32)	21(32.81)			
C allele		52/272(22.43)	49/128(31.25)	9.626	0.002	2.002(1.286—3.119)
		Size(≥3 cm) (72 patients)	Size(<3 cm) (128 patients)	X^2^ value	*P* value	
rs1333040	CC	8(11.11)	11(8.59)			
	CT	6 (8.33)	57(44.53)	28.355	0.000^*^	
	TT	58(80.56)	60(46.88)			
C allele		22/144(15.28)	79/256(30.86)	7.358	0.007	2.020(1.207—3.380)
rs6475606	CC	7(9.72)	12(9.37)			
	CT	23(31.94)	40(31.25)	0.021	0.989	
	TT	42(58.33)	76(59.38)			

*P < 0.000

## Discussion

In this study, we aim to explore CDKN2BAS genetic relationship with Chinese han nationality of IAs. We provided evidence that the variants rs1333040 and rs6475606 were associated with IAs, in agreement with the results of Hashikata et al. and Foroud et al., respectively. Hashikata et al. based on previous results of Genome-wide association studies(GWAS) confirmed rs1333040 association with familial and sporadic IAs in Japanese patients, their study included 419 sporadic IA cases and 408 control subjects; Foroud et al. utilized the similar approach to investigate the role of rs6475606 in 1095 IA cases and 1286 controls, as the size of the sample, our results with theirs differ markedly.

Rs3217992, rs974336, rs3217986, rs1063192 are the highest mutation rate locus of CDKN2BAS gene in Chinese population. We can learn from the Table 4 that the four sites genotype frequency and allele frequency were close in the experimental group and the control group. Statistical results show that these loci loss correlation with sporadic IAs of China(all *P* values > 0.05), rs6475606, rs1333040 and rs10757272 are the highest positive sites correlation with IAs in overseas study,reflect the Japan and the United States population, rs1333040 and rs6475606 were also replicated, but no association of the variant rs10757272 with sporadic IAs was found in our study, the conflicting results mainly root in genetic heterogeneity in different ethnic populations, additionally, the presence of genetic heterogeneity may be relevant in a bias caused by variation in the prevalence of a positive family history between populations, because familial IAs have a higher risk than sporadic IAs, negative results for this SNP do not exclude CDKN2BAS SNP plays an important role in aneurysms. The other four loci selected in this study which is the highest mutation rates in Chinese population were unrelated to aneurysms, among previous studies, a research thinks disease-causing mutations may be located in MAF < 0.05, and influence the formation of IAs. We also detected statistically significant correlations between CDKN2BAS SNPs and hemorrhage and aneurysm size, rs1333040_C did not work in aneurysm formation, but it could reduce the occurrence of hemorrhage and it was the risk factor of IAs with size < 3 cm; the rs6475606_C could reduce the occurrence of IAs, but the impact of it on hemorrhage and size was not clear, the reason may be related to the research errors.

The sequence of CDKN2BAS gene is so long that it will be very difficult to find the exact loci associated with IA. Rs6475604, rs1333040, rs10757272 associated with IA come from GWAS mostly, these loci are not for more research, and lack of functional studies. IA is a complex genetic disease, the interaction between genes and genes as racial differences will affect the results of our study. CDKN2BAS belongs to long noncoding RNA(lncRNA) and affects the surrounding gene expression indirectly play a role in the disease, Bai Y research results show that CDKN2BAS can regulate the expression of CARD8 level, which affects the progress of atherosclerosis, compare the RNA in aneurysm tissue and normal cerebrovascular, hundreds of lncRNA exists differences, we can’t help but question: would CDKN2BAS cause IA formation? Or CDKN2BAS associated with IA could be caused by other lncRNA [2, 15, 19].

The current generally accepted IA is the result of the dual role of genetic and environmental factors, screening finds a large number of genes associated with IA, but these genes effect on IA is too low, thus known that the influence of environmental factors on the disease may be greater, CDKN2BAS gene polymorphism correlated with IA in other population, but has nothing to do with the Chinese people, there is a hypothesis: the environment makes IA more susceptible to CDKN2BAS in other population, although rs3217992, rs974336, rs3217986 and rs1063192 are the highest mutation rate loci of CDKN2BAS gene in Chinese population, influenced by environmental factors, they without associated with IA [11].

In addition to the above mentioned several assumptions, our study associated with the degree and distribution of linkage disequilibrium [1]. Research work in susceptibility genes of IA did not make substantive progress, scholars have been staying at the screening level, but many of genes repeatability is poor, the author considers not all IA are related to gene mutation, genetic, environmental and perhaps many other factors involved in the IA progress, it needs us to further research.

## Conclusions

Our study showed that CDKN2BAS polymorphism might be associated with intracranial aneurysms in Chinese population. Additional studies are needed to confirm our finding.

## Abbreviations

ACA: Anterior cerebral artery
ACoA: Anterior communicating artery
CI: Confidence intervals
CTA: Computed tomography angiography
DSA: Digital subtraction angiography
GWAS: Genome-wide association study
IA: Intracranial aneurysm
ICA: Internal carotid artery
LDR: Ligase detection reaction
lncRNA: long noncoding RNA
MAF: Minor allele frequency
MCA: Middle cerebral artery
MRA: Magnetic resonance angiography
MRI: Magnetic resonance imaging
OR: Odds ratio
PCA: Posterior cerebral artery
PCoA: Posterior communicating artery
PCR: Polymerase chain reaction
SNP: Single nucleotide polymorphism
